# Leveraging Integrated RNA Sequencing to Decipher Adrenomedullin’s Protective Mechanisms in Experimental Bronchopulmonary Dysplasia

**DOI:** 10.3390/genes15060806

**Published:** 2024-06-19

**Authors:** Subarna Palit, Amrit Kumar Shrestha, Shyam Thapa, Sandra L. Grimm, Cristian Coarfa, Fabian Theis, Lukas M. Simon, Binoy Shivanna

**Affiliations:** 1 TUM School of Life Sciences Weihenstephan, Technical University of Munich, 85354 Freising, Germany; 2 Division of Neonatology, Department of Pediatrics, Texas Children’s Hospital, Baylor College of Medicine, Houston, TX 77030, USAshivanna@bcm.edu (B.S.); 3 Department of Molecular and Cellular Biology, Baylor College of Medicine, Houston, TX 77030, USA; 4 Institute of Computational Biology, Helmholtz Zentrum München, 85764 Neuherberg, Germany; 5 Department of Mathematics, Technical University of Munich, 85748 Garching, Germany; 6 Therapeutic Innovation Center, Baylor College of Medicine, Houston, TX 77030, USA

**Keywords:** bronchopulmonary dysplasia, adrenomedullin, RNA-seq, natural killer cells

## Abstract

Bronchopulmonary dysplasia (BPD) is a chronic lung disease commonly affecting premature infants, with limited therapeutic options and increased long-term consequences. Adrenomedullin (*Adm*), a proangiogenic peptide hormone, has been found to protect rodents against experimental BPD. This study aims to elucidate the molecular and cellular mechanisms through which *Adm* influences BPD pathogenesis using a lipopolysaccharide (LPS)-induced model of experimental BPD in mice. Bulk RNA sequencing of *Adm*-sufficient (wild-type or *Adm*^+/+^) and *Adm*-haplodeficient (*Adm*^+/−^) mice lungs, integrated with single-cell RNA sequencing data, revealed distinct gene expression patterns and cell type alterations associated with *Adm* deficiency and LPS exposure. Notably, computational integration with cell atlas data revealed that *Adm*-haplodeficient mouse lungs exhibited gene expression signatures characteristic of increased inflammation, natural killer (NK) cell frequency, and decreased endothelial cell and type II pneumocyte frequency. Furthermore, in silico human BPD patient data analysis supported our cell type frequency finding, highlighting elevated NK cells in BPD infants. These results underscore the protective role of *Adm* in experimental BPD and emphasize that it is a potential therapeutic target for BPD infants with an inflammatory phenotype.

## 1. Introduction

Bronchopulmonary dysplasia (BPD) is a chronic lung disease that primarily affects premature infants, particularly those born before 28 weeks of gestation [1,2,3]. The burden of BPD lies in its increased prevalence, significant morbidity and mortality rates among preterm infants, lack of specific therapies, as well as its potential long-term respiratory and developmental consequences [4,5,6,7,8]. The pathogenesis of BPD is complex and multifactorial, involving both prenatal and postnatal factors [2,9,10,11]. Prenatal factors include intrauterine inflammation, intrauterine growth restriction, and genetic predispositions. Postnatally, factors such as mechanical ventilation, oxygen toxicity, infections, and inflammation contribute to lung injury and impaired alveolar development. The vascular hypothesis of BPD suggests that impaired vascular development and abnormal pulmonary angiogenesis play a critical role in the pathogenesis of the disease [12,13]. Premature birth disrupts the normal development of the pulmonary vasculature, leading to decreased vascular growth and altered vascular structure. This disruption results in decreased perfusion, impaired oxygen delivery, and increased pulmonary vascular resistance, contributing to lung injury and impaired alveolarization. Therefore, targets that promote lung endothelial cell health in the developing lungs can potentially prevent and mitigate BPD in premature infants.

Adrenomedullin (*Adm*) is a peptide hormone involved in various physiological processes such as vasodilation, regulation of blood pressure, and fluid balance. Kitamura et al. first discovered it in 1993 as a novel hypotensive peptide isolated from human pheochromocytoma, a tumor of the adrenal medulla [14]. *Adm* is derived from a larger precursor molecule called preproadrenomedullin, which is cleaved to produce the active form [15]. In humans, it is encoded on chromosome 11 [16], and in mice, it is encoded on chromosome 7 [17]. The peptide acts through multiple receptors, including the calcitonin receptor-like receptor (CLR) and receptor activity-modifying proteins (RAMPs) [18]. Upon binding to its receptors, *Adm* activates intracellular signaling pathways, such as cAMP production, leading to vasodilation and other physiological responses. The peptide is widely expressed in highly vascularized tissues and organs, including the cardiovascular system, lungs, kidneys, and brain since the gene encoding *Adm* is expressed notably in endothelial and vascular smooth muscle cells [19,20]. Its expression is up-regulated in response to various stimuli such as hypoxia, inflammation, and mechanical stress [19,21]. *Adm* has been implicated in several pathological conditions, including cardiovascular diseases [20,22,23,24,25], pulmonary disorders [26,27,28,29,30], gastrointestinal disorders [31,32], sepsis [33,34,35,36], cancer [20,32,37,38], and disorders associated with pregnancy [39,40,41,42]. Since *Adm* is highly enriched in the lungs, its expression is influenced by inflammation, and the peptide plays a vital role in maintaining endothelial cell homeostasis [43,44,45]; *Adm* may play a pathogenic role in inflammatory lung disorders such as BPD.

Studies involving *Adm* in BPD have focused on understanding its role in the pathogenesis of the disease and its potential as a biomarker or therapeutic target. Others and ourselves have highlighted the protective role of *Adm* in preclinical BPD studies. Insults such as hyperoxia and lipopolysaccharide (LPS) alter *Adm* expression and signaling in the developing lungs of rodents [44,45,46,47,48]. Further, BPD infants also have higher *Adm* expression compared to non-BPD infants [49]. Yet, decreased *Adm* expression or signaling causes or augments BPD phenotype [45,47,48], and *Adm* peptide treatment mitigates experimental BPD [47]. These findings emphasize the protective role of *Adm* in neonatal lungs and its potential to serve as a biomarker and therapeutic target for BPD infants. Despite these discoveries, the intricate cellular and molecular mechanisms through which *Adm* exerts its protective effects on lung development, repair, and injury remain unclear. Identifying these mechanisms holds promise for developing precise prediction models and therapies for the disease. Therefore, we performed unbiased bulk RNA sequencing of the lungs from *Adm*-sufficient and haplodeficient mice in a well-established LPS-induced model of experimental BPD [48] and integrated bulk RNA sequencing results with state-of-the-art single-cell lung atlas studies to resolve how *Adm* protects neonatal lungs against chronic injury.

## 2. Materials and Methods

### 2.1. Animals

The Institutional Animal Care and Use Committee of Baylor College of Medicine approved this study (Protocol # AN-5631). Dr. Kathleen Caron (University of North Carolina, Chapel Hill, NC, USA) provided us with the *Adm* haplodeficient (*Adm*^+/−^) mice on a 129/SvEv background, as previously documented [23]. To obtain *Adm*^+/−^ mice on a C57BL/6J background for our studies, we performed 12 generations of backcrossing of the original *Adm*^+/−^ mice with C57BL/6J wild-type mice (stock no. 000664; The Jackson Laboratory, Bar Harbor, ME, USA). We utilized time-pregnant mice raised in our animal facility for the studies. Since *Adm*^−/−^ mice are embryonically lethal, we used *Adm*^+/−^ mice for our studies. We confirmed the genotype of *Adm*^+/−^ mice through both genotyping and real-time RT-PCR analysis of lung tissues for *Adm* expression.

### 2.2. LPS Treatment

For the LPS treatment, we administered intraperitoneal injections of 6 mg/kg of *Escherichia coli* O55:B5 LPS (Sigma-Aldrich, St. Louis, MO, USA; L2880) or an equivalent volume of PBS to both wild-type (WT) or *Adm*^+/+^ and *Adm*^+/−^ mice once daily for three days on postnatal days (Ps) 3–5 [48].

### 2.3. Lung Tissue Extraction, RNA Isolation, and Bulk RNA Sequencing

At P5, 6 h after the third dose of PBS or LPS, we extracted lung tissue from the study animals, immediately snap-froze it in liquid nitrogen, and stored it at −80 °C for subsequent RNA isolation. Total lung RNA was isolated from the lung tissues of mice exposed to PBS or LPS (*n* = 3/genotype/exposure) and purified using the Direct-zol RNA MiniPrep kit (Zymo Research, Irvine, CA, USA; R2052). The Genomic and RNA Profiling Core at Baylor College of Medicine first conducted sample quality checks using the NanoDrop spectrophotometer (Thermo Fisher Scientific, Waltham, MA, USA), Invitrogen Qubit 2.0 quantitation assay (Thermo Fisher Scientific), and Agilent Bioanalyzer (Agilent Technologies, Santa Clara, CA, USA). RNA-seq libraries were next generated using the NEBNext Ultra II Directional RNA-seq kit (New England Biolabs, Ipswich, MA, USA) (p/n E7760) with rRNA depletion (p/n E7400), according to the manufacturer’s instructions. RNA-seq libraries were sequenced on an Illumina NovaSeq 6000 sequencer to around 85 million reads per sample. The RNA-seq data were mapped using HISAT2 (v2.1.0) to the mouse genome build UCSC mm10. Aligned reads were counted against gene model annotation (Gencode) to obtain expression values by using Feature Counts (v1.6.4).

### 2.4. Data Transformations for Exploratory Analysis

All analyses were performed on protein-coding genes. For inter-sample normalization at the gene level, the R package edgeR [50] was employed, utilizing the weighted trimmed mean (TMM) method. Subsequently, relative scaling factors for the libraries were computed, and normalized counts per million (CPM) values were derived within the edgeR framework. Genes with very low counts across all the libraries were also removed prior to downstream analysis.

### 2.5. Exploratory Analysis of RNA Expression

Exploratory data analysis was performed on the log-transformed expression values. Principal component analysis (PCA) served as a visualization tool to examine the clustering patterns in our dataset. Features with no detectable signal across all samples were excluded from consideration to prevent zero variance issues. The remaining 15,885 (72%) genes were subjected to PCA using the prcomp implementation in R (v4.2.1).

### 2.6. Differential Expression Analysis

RNA-seq data were subjected to linear modeling using empirical Bayes statistics from the limma package in R to detect differentially expressed genes [51]. Specifically, our models incorporated effects for treatment (LPS or PBS) within each genotype (WT and *Adm*^+/−^ mice), enabling the detection of differential treatment effects between genotypes. We applied the Benjamini–Hochberg correction to control the false discovery rate (FDR) among selected significant genes. Genes were considered statistically significant for individual variables (i.e., differentially expressed in *Adm*^+/−^ over WT; between PBS and LPS treatment) if their adjusted *p*-value was below 0.05 and if the fold change exceeded 0.5 times. Interaction effects were computed by fitting a 2-factor model on the condition (WT, *Adm*^+/−^) and treatment (LPS, PBS). Preference was given to genes with significant differential effects on treatment and condition combined.

### 2.7. Cell Type Deconvolution Analysis

Following our previous work, we performed cell-type deconvolution analysis [52,53,54]. Bulk RNA-seq differential expression results were subjected to enrichment analyses using single-cell RNA-seq derived cell type markers to discover changes in cell type frequency. Enrichment analysis was carried out using the Enrichr R package [55] on Tabula Muris [56] cell types. To further validate the differentially enriched lung cell types, we cross-referenced our findings with a publicly available murine lung cell atlas called LungMAP (www.lungmap.net; accessed on 13 June 2023). LungMAP catalogs RNA expression from 95,658 lung cells across 8 timepoints from embryonic day 16.5 to postnatal day 28, identifying 37 well-defined and 3 novel (Sox9+/Id2+ DEP, EPC, and PMP) lung cell types [57]. The top 10 cell type markers with differential effects in *Adm*^+/−^ compared to WT were visualized by extracting their average RNA expression by lineage and cell types in the LungMAP atlas. Briefly, genes that were positively enriched in *Adm*^+/−^ vs. WT mice were selected as candidates based on having (i) higher expression in *Adm*^+/−^ mice exposed to LPS vs. PBS and (ii) higher expression in LPS-treated *Adm*^+/−^ vs. WT mice. Additionally, candidate genes needed to be lung markers [56], have expression above the 15th percentile of the data, and show significant interaction *p*-value (finding genes with differential effects to LPS treatment in WT or *Adm*^+/−^). All analyses with the LungMAP dataset were performed using the default Scanpy workflow (v1.9.1) [58].

### 2.8. KEGG Pathway Enrichment Analysis

To gain insights into the biological pathways associated with the differentially expressed genes (DEGs), we performed a KEGG enrichment analysis. Genes were categorized into seven distinct groups based on their unique expression profiles between WT and *Adm*^+/−^ mice in response to LPS treatment. Each group of DEGs was subjected to KEGG analysis using the Enrichr R package [55] to identify significantly enriched pathways. We used the Enrichr R package for the enrichment analysis due to its robust statistical methods and comprehensive KEGG database integration [59]. Enriched pathways with adjusted *p*-values less than 0.25 and containing 5 or more representative genes were considered significant. The complete lists of DEGs for each category and their corresponding KEGG pathway enrichment results are provided in Supplementary Table S5. Additionally, Supplementary Figure S1 shows the top enriched pathways for each gene category, with gene counts and *p*-values indicated.

### 2.9. Analysis of BPD Cohort

Statistical analyses were performed using the Bioconductor limma package in R [51]. Raw RNA-seq counts were downloaded from the Gene Expression Omnibus [60] with accession GSE156028, and standard pre-processing steps were followed. Patient characteristics are reported in Supplementary Table S4. Gene expression normalizations were performed using the TMM method, and low-count genes were removed before further downstream analysis. Differential genes were identified with the edgeR package in R [50], and differential expression was defined as a Benjamini Hochberg FDR < 0.05. In order to test for enrichment of NK cell markers within the differential log fold changes between infants with and without BPD, we applied the fgseaMultilevel function from the fgsea R package [61]. Additionally, the empirical cumulative distribution function (stat_ecdf from the R ggplot2 package [62]) plot was utilized to assess the distribution of observed differential expression fold changes in top NK marker genes.

### 2.10. Code Availability

The code to reproduce our analyses will be available on GitHub: https://github.com/lkmklsmn/adm_paper (accessed on 13 June 2024).

### 2.11. Data Availability

The bulk RNA sequencing dataset generated in this study is available at the National Center for Biotechnology Information Gene Expression Omnibus (GEO) under the accession number GSE264612.

## 3. Results

### 3.1. Bulk RNA-Seq Analysis Reveals Transcriptomic Alterations in Response to LPS

**Figure 1:**
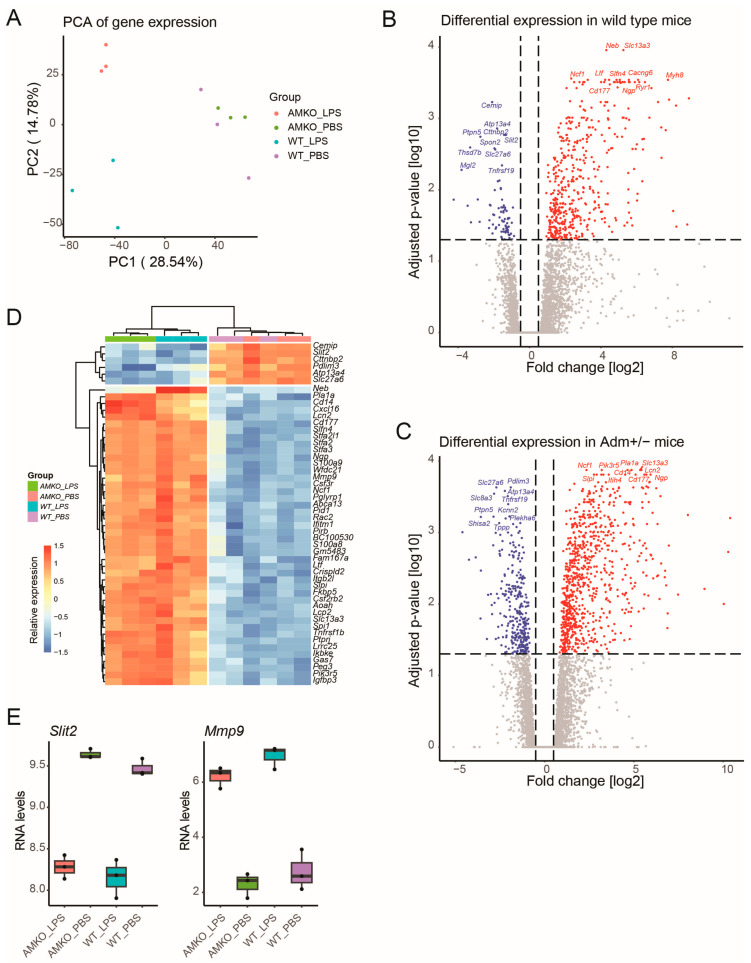
Bulk RNA-seq analysis reveals transcriptomic alterations in response to LPS. (**A**) Principal component analysis (PCA) projection of samples across experimental conditions based on protein-coding genes demonstrated clear separations between samples from different conditions. Two independent regressions were applied comparing LPS- to PBS-treated samples in WT (**B**) and *Adm*^+/−^ (**C**) mice. Genes with significant up- and down-regulation are colored red and blue, respectively. Genes without significant differential expression are colored grey. (**D**) The heatmap displays the relative expression of the top 50 most differentially expressed genes across both comparisons. (**E**) Boxplot shows up and down-regulation of representative genes *Mmp9* and *Slit2*, respectively, upon LPS exposure in both genotypes. Boxplots show the median value in the corresponding group, the extreme values, and the interquartile range (IQR). Whiskers indicate 1.5 IQR of the lower and upper quartiles. Black dots represent outliers defined as being more than 1.5 times the IQR above the third quartile or below the first quartile.

To investigate the role of *Adm* in LPS-induced experimental BPD in mice, bulk RNA sequencing data were generated from *Adm*-haplodeficient (*Adm*^+/*−*^) and WT (*Adm*^+/*+*^) mice using whole lung tissue in response to LPS treatment or control PBS in triplicates. Unsupervised dimension reduction of 12 samples across four groups based on 15,885 unique protein-coding genes using principal component analysis revealed distinct clustering by condition, indicating reproducible and high-quality data (Figure 1A). Moreover, the first principal component demonstrated clear separation between animals exposed to LPS from controls for both genotypes. The second principal component further stratified LPS-exposed *Adm*^+/*−*^ from WT samples, indicating that the major source of variation differentiated the LPS response rather than technical variation in *Adm*^+/*−*^ mice compared to WT mice.

Differential expression analysis was performed by independently comparing LPS exposure to controls in *Adm*^+/*−*^ and WT mice. In WT mice, 542 genes (Table S1, Figure 1B), and in *Adm*^+/*−*^ mice, 1139 genes were differentially expressed (Table S2, Figure 1C) at absolute log fold changes above 0.5 and adjusted *p*-value below 0.05. The heatmap shows the z-transformed expression of the top 50 genes differentially regulated in response to LPS in both genotypes (Figure 1D). For example, genes *Slit2* and *Mmp9* were significantly down-regulated and up-regulated, respectively, in response to LPS treatment across both genotypes (Figure 1E).

### 3.2. Published RNA and Protein Expression Experiments Validate Bulk RNA-Seq Data

**Figure 2:**
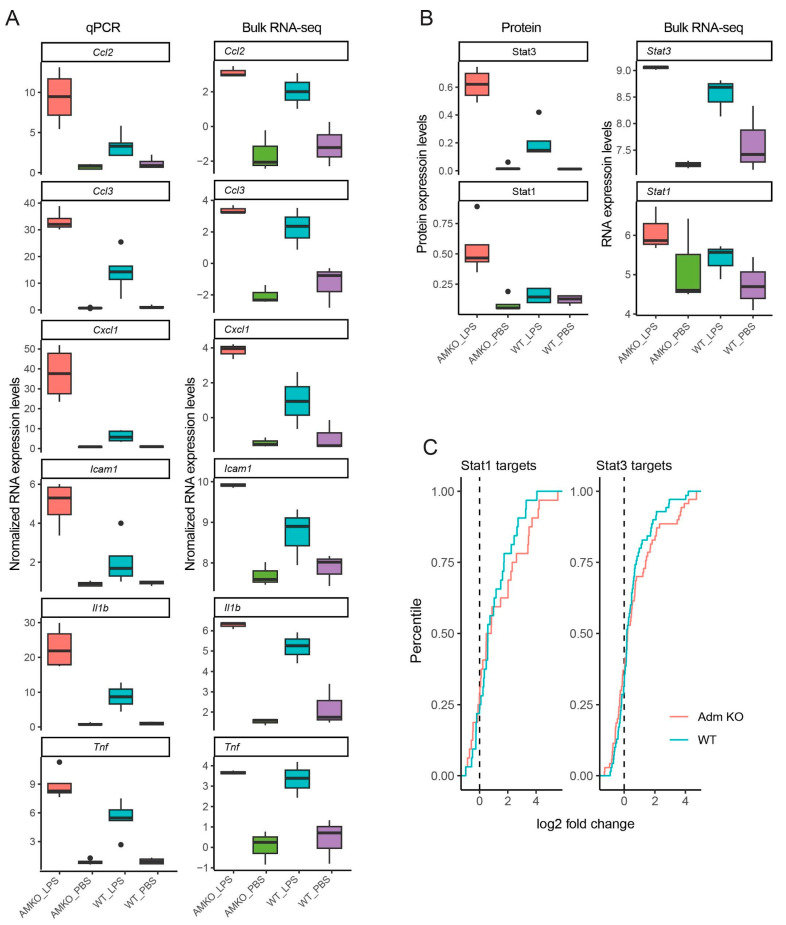
Published RNA and protein expression experiments validate bulk RNA-seq data: (**A**) Boxplots show quantification of RNA levels based on qPCR (left) and bulk RNA-seq (right) across select genes with increased up-regulation in *Adm*^+/*−*^ compared to WT. (**B**) Boxplots show quantification of protein expression (left) and RNA expression based on bulk RNA-seq (right) for transcription factors Stat1 and Stat3. For both (**A**) and (**B**), boxplots show the median value in the corresponding group, the extreme values, and the interquartile range (IQR). Whiskers indicate 1.5 IQR of the lower and upper quartiles. Black dots represent outliers defined as being more than 1.5 times the IQR above the third quartile or below the first quartile. n = 4 to 5 mice per group for real-time RT-PCR and protein expression studies. n = 3 mice per group in bulk RNA-seq study. (**C**) Empirical cumulative density plots show expression fold change (*x*-axis) of Stat1 (left) and Stat3 (right) targets in *Adm*^+/*−*^ (red) and WT mice (blue).

To validate the bulk RNA-seq data generated in this study, we compared the results of the RNA-seq data with the RNA and protein expression data from our previous study in *Adm*^+/*−*^ and WT mice exposed to LPS or PBS [48]. In the previous study, the RNA and protein expression changes for select genes were quantified using quantitative polymerase chain reaction (qPCR) and immunoblotting studies in *Adm*^+/*−*^ and WT mice using the same LPS-induced experimental BPD model [48]. Increased up-regulation of genes *Ccl2*, *Ccl3*, *Cxcl1*, *Icam1*, *Il1b*, and *Tnf* in *Adm*^+/*−*^ compared to WT mice was confirmed by the previous qPCR study results (Figure 2A) [48]. Furthermore, increased up-regulation of transcription factors Stat1 and Stat3 at the protein level in the prior study [48] was also mirrored at the RNA level, as observed in our bulk RNA-seq data (Figure 2B). Moreover, Stat1 and Stat3 targets were significantly enriched in genes up-regulated in response to LPS in WT mice (Stat1 enrichment *p*: 0.00049; Stat3 enrichment *p*: 0.0041, Figure 2C). This enrichment was further exacerbated in *Adm*^+/*−*^ mice (Stat1 enrichment *p*: 1.75 × 10^−5^; Stat3 enrichment *p*: 9.1 × 10^−5^). Taken together, these results validate the differential expression patterns observed in our newly generated bulk RNA-seq data.

### 3.3. Preferential Regulation Analysis Reveals Cell Type Frequency Changes Specific to Adm^+/−^ Mice

**Figure 3:**
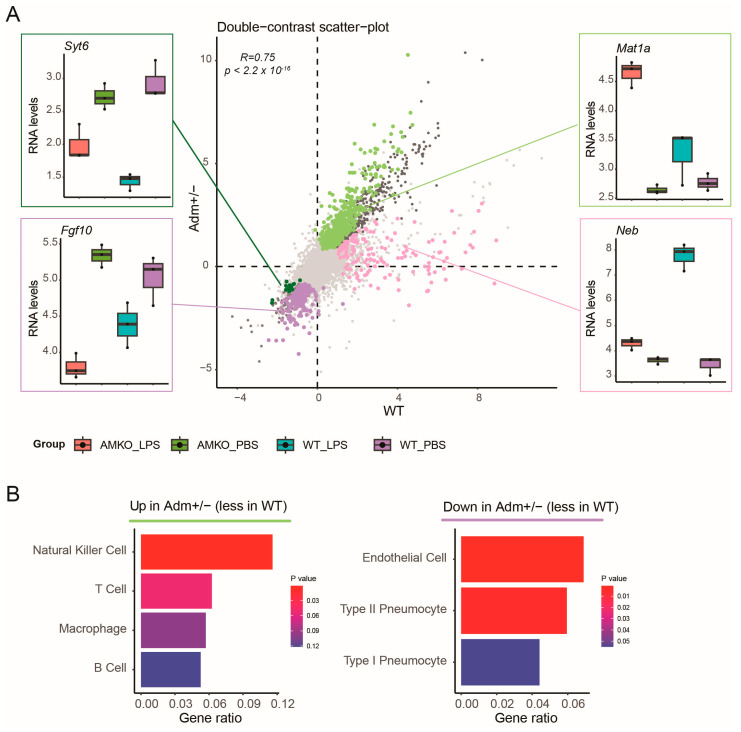
Preferential regulation analysis reveals cell type frequency changes specific to *Adm*^+/*−*^ mice: (**A**) Scatter plot shows LPS induced log fold changes in WT (*x*-axis) and *Adm*^+/*−*^ mice (*y*-axis). Colors indicate genes with differential response to LPS treatment between *Adm*^+/*−*^ and WT mice. Boxplots highlight representative genes that responded differently to LPS exposure in *Adm*^+/*−*^ compared to WT. Genes without significant differential expression are colored grey. Boxplots show the median value in the corresponding group, the extreme values, and the interquartile range (IQR). Whiskers indicate 1.5 IQR of the lower and upper quartiles. Black dots represent outliers defined as being more than 1.5 times the IQR above the third quartile or below the first quartile. (**B**) Barplots visualize gene ratio (*x*-axis) derived from enrichment tests for lung cell types (*y*-axis). Cell types were considered significant if the adjusted *p*-value was less than 0.25. Colors represent statistical significance.

Next, we assessed the differences in LPS response in *Adm*^+/−^ mice compared to WT mice. While expression fold changes in the LPS response in *Adm*^+/−^ mice compared to WT mice correlated significantly on a global level (Figure 3A, Rho 0.75, *p* < 2.2 × 10^−16^), numerous genes showed preferential up- or down-regulation in one of the two contrasts. Therefore, we performed preferential regulation analysis by stratifying all 15,885 genes into seven categories based on the differential expression patterns: (1) 14,591 genes without differential expression in either genotype; (2) 64 genes significantly down-regulated in both genotypes; (3) 323 genes significantly up-regulated in both genotypes; (4) 499 genes up-regulated in *Adm*^+/−^ mice without significant change in WT mice; (5) 253 genes down-regulated in *Adm*^+/−^ mice without significant change in WT mice; (6) 141 genes significantly up-regulated in WT mice without significant change in *Adm*^+/−^ mice; (7) 14 genes down-regulated in WT mice without significant change in *Adm*^+/−^ mice.

To assess the molecular pathways underlying these expression patterns, we performed a gene set enrichment analysis (Supplementary Figure S1). Genes showing no differential expression in either genotype (category 1) were excluded from the analysis. The remaining six gene sets were subjected to enrichment analysis using the KEGG database. No significant enrichment was identified for category 2. However, genes significantly up-regulated in both genotypes (category 3) were enriched in infection-related genes set including “Cytokine-cytokine receptor interaction” (adjusted *p*-value: 9.12 × 10^−10^).

Among genes showing preferential regulation, *Syt6* displayed stronger down-regulation upon LPS treatment in WT compared to *Adm*^+/−^ mice. The gene *Neb* showed stronger up-regulation upon LPS treatment in WT compared to *Adm*^+/−^ mice (Figure 3A). However, neither of the genes were enriched in any significant genesets. Moreover, no significant pathway enrichment was identified for genes in category 7. Category 6, which contained genes significantly up-regulated in WT mice without being differential in *Adm*^+/−^, exhibited differential regulation in two pathways, including the “Calcium signaling pathway” (adjusted *p*-value: 0.24). However, these were not pursued further due to their borderline significance.

In contrast, genes up-regulated in *Adm*^+/−^ mice without significant change in WT mice (category 4) were enriched in several inflammation-relevant signaling pathways, including TNF, NF-kappa B, and JAK-STAT (adjusted *p*-value: <1 × 10^−7^). For example, the gene *Mat1a* was strongly up-regulated in response to LPS in *Adm*^+/−^ mice but only moderately altered in WT mice. Genes down-regulated in *Adm*^+/−^ mice without significant change in WT mice (category 5) were enriched in Ras, Calcium, and MAPK signaling pathways (adjusted *p*-value: <0.25). For example, *Fgf10*, a member of all these three pathways, showed preferential down-regulation in *Adm*^+/−^ upon LPS treatment compared to WT (Figure 3A).

We subsequently assessed if the underlying gene expression changes reflected differences in cell type composition in the lungs using cell type deconvolution analysis. Towards this end, we applied gene set enrichment analysis using cell type marker annotation from the Tabula Muris resource [56], restricted to cell type markers from the lung. Enrichment analysis revealed marker genes characterizing natural killer (NK) cells to be preferentially up-regulated in *Adm*^+/−^ compared to WT (Figure 3B, FDR < 0.05), indicating a higher presence or activity of NK cells in *Adm*^+/−^. In contrast, marker genes associated with Type II pneumocytes and endothelial cells were significantly enriched among genes that were preferentially down-regulated in *Adm*^+/−^ compared to WT mice (Figure 3B, FDR < 0.05), potentially speculating their inactivity. No significant lung cell enrichment was observed for genes that were preferentially altered in WT mice. These results suggest that *Adm*^+/−^ mice showed a reduced frequency of type II pneumocytes and endothelial cells while increasing the frequency of NK cells upon LPS exposure compared to WT mice.

### 3.4. Integration of Lung Cell Atlas Reveals Alterations in Cell Type Markers

Given that three cell types were significantly enriched amongst preferentially altered genes, we subsequently focused our analysis on the expression of NK cell, endothelial cell, and type II pneumocyte signatures. We cross-referenced our findings with the murine single-cell lung atlas resource called LungMAP [57]. LungMAP catalogs RNA expression of 95,658 lung cells across lung development, cataloging 40 different cell types. Thus, LungMAP provides robust marker genes for each lung cell type.

**Figure 4:**
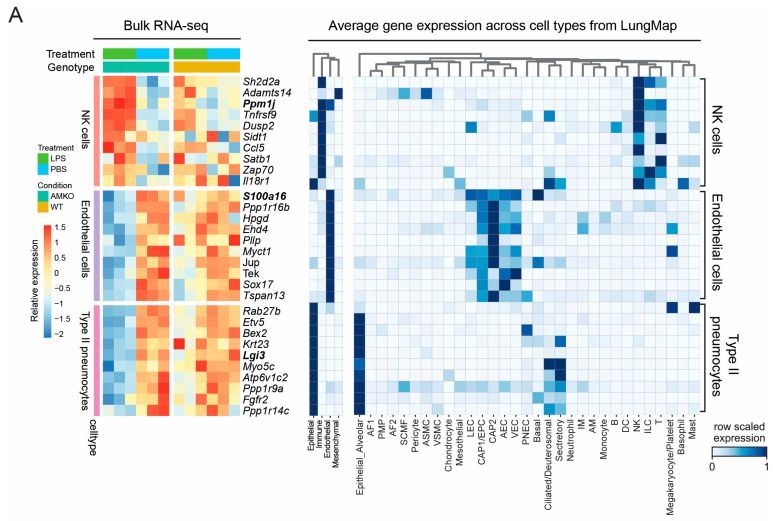

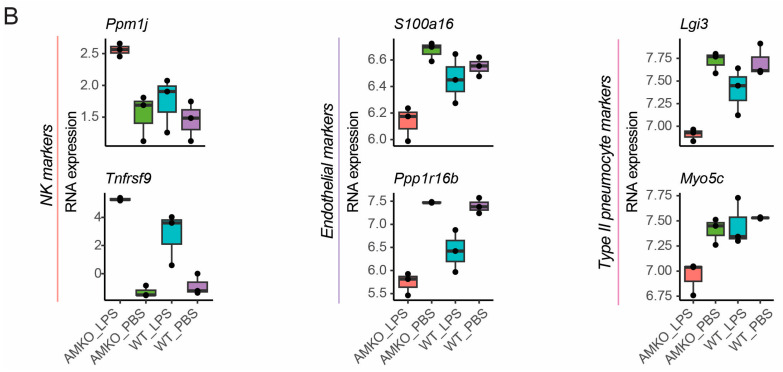
Integration of lung cell atlas reveals alterations in cell type markers: (**A**) The heatmap on the left visualizes the relative expression of the top 10 cell type markers from the three implicated lung cell types with differential activity in the bulk RNA-seq. Blue and red colors represent low and high expression values, respectively. The heatmaps on the right show the average expression of the same set of marker genes aggregated by lineage and cell types in the LungMAP single-cell atlas. White and blue colors represent low and high expression values, respectively. (**B**) Boxplots of RNA expression levels for representative markers from the three cell types with differential activity. Boxplots show the median value in the corresponding group, the extreme values, and the interquartile range (IQR). Whiskers indicate 1.5 IQR of the lower and upper quartiles. Black dots represent outliers defined as being more than 1.5 times the IQR above the third quartile or below the first quartile.

Restricting the analysis to the top 10 most preferentially expressed cell type markers for NK cells, type II pneumocytes, and endothelial cells showed coordinate expression alterations in the *Adm*^+/*−*^ mice with decreased signal in the WT mice (Figure 4A). Visualization of the average expression of these genes in the LungMAP atlas demonstrated cell type specificity. For example, two NK cell type marker genes, *Ppm1j* and *Tnfrsf9*, were strongly up-regulated in *Adm*^+/*−*^ mice, while expression changes in WT mice showed weaker up-regulation. Endothelial cell type markers *S100a16* and *Ppp1r16b*, on the other hand, showed stronger down-regulation in *Adm*^+/*−*^ compared to WT mice. Similar patterns were observed for Type II pneumocyte markers *Lgi3* and *Myo5c* (Figure 4B). Taken together, these results are suggestive of the fact that the expression changes observed in *Adm*^+/*−*^ mice reflect changes in actual cell type frequency of NK cells, type II pneumocytes, and endothelial cells, indicating that in patients who develop BPD, it is likely that the lung selectively either recruits or depletes several key cell types otherwise required for maintaining lung health.

### 3.5. RNA Expression Profile of BPD Patients Reflects Increase in NK Cells

**Figure 5:**
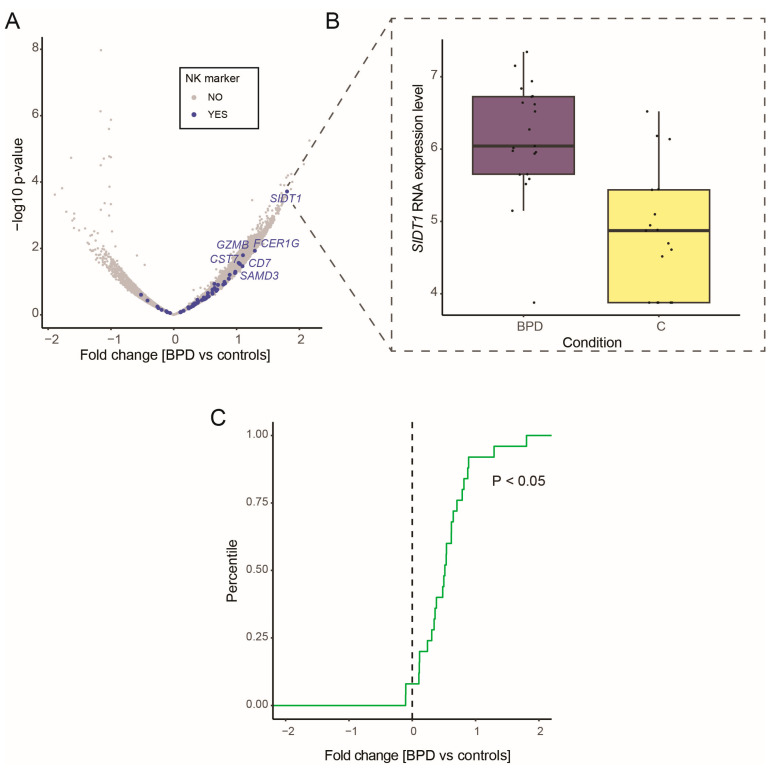
RNA expression profile of BPD patients reflects an increase in NK cells: (**A**) Volcano plot shows differential gene expression of tracheal aspirate samples from infants with and without BPD diagnosis. The highlighted genes in blue are NK cell lung markers sourced from Tabula Muris. (**B**) Boxplot shows increased expression of *SIDT1* in BPD patients. Boxplot shows the median value in the corresponding group, the extreme values, and the interquartile range (IQR). Whiskers indicate 1.5 IQR of the lower and upper quartiles. Black dots represent data points. (**C**) The empirical cumulative density plot displays the distribution of fold changes for NK cell markers in BPD patients compared to controls.

Having discovered gene expression changes reflecting differences in the lung cell type composition in *Adm*^+/*−*^ mice in our LPS-induced experimental BPD mouse model, we examined whether there were similar changes in a human BPD cohort [63]. The cohort contained mRNA expression profiles from tracheal aspirates of 38 infants, of which 17 were neonates without lung disease, and 21 infants were diagnosed with BPD. Given our findings from the *Adm*^+/*−*^ experimental BPD mouse model, we hypothesized to observe an increase in NK cells in BPD patients compared to controls. Therefore, we performed differential gene expression analysis between BPD and controls (Table S3). Several NK cell markers were up-regulated (Figure 5A). For example, NK marker *SIDT1* was significantly up-regulated in BPD patients compared to controls (FDR < 0.05, Figure 5B). Next, we statistically assessed the enrichment of NK cell, type II pneumocyte, and endothelial cell markers within the differentially expressed genes. Significant enrichment of NK cell markers was observed within genes up-regulated in BPD (enrichment *p*-value < 0.05, Figure 5C). However, no significant enrichment was observed for endothelial cell and type II pneumocyte markers. These results are in agreement with the expression changes observed in the mouse *Adm*^+/*−*^ samples upon LPS exposure, and we hypothesize a potential role of NK cell type in BPD pathogenesis.

## 4. Discussion

This research utilized unbiased, high-throughput, and state-of-the-art bioinformatic methods to understand the intricate molecular and cellular mechanisms through which *Adm* influences the outcomes of LPS-induced experimental BPD in mice. We found that *Adm* influences lung inflammation and injury via distinct molecular pathways and lung cell types. The results lay the groundwork for future mechanistic and therapeutic studies aimed at preventing and mitigating BPD in preterm infants.

*Adm* has emerged as a crucial regulator of neonatal lung injury, as evidenced by studies in newborn rodents [45,47,48] and primary human fetal lung endothelial cells [44,45,46]. To delve deeper into the role of *Adm* in neonatal lung biology, we used an LPS-induced model of experimental BPD [48], given that postnatal sepsis is one of the major causes of human BPD [64,65,66] and that LPS is commonly used to model sepsis in neonatal rodents [48,67,68,69]. Additionally, the cellular and molecular mechanisms through which *Adm* regulates sepsis-induced neonatal lung injury are unclear. Furthermore, *Adm* is an angiogenic hormone, and lung vascular development and endothelial cell signaling are critical for alveolarization; disruptions in endothelial cell homeostasis can impede this process and cause alveolar simplification, a hallmark of BPD [13,70]. Thus, we conducted a genome-wide transcriptional study using an established LPS-induced experimental BPD model to pinpoint the genes and pathways affected by LPS in general and specifically by *Adm* to gain deeper insights into how *Adm* modulates LPS-induced neonatal lung injury. While RNA-Seq analyses at a single time point may not capture all deregulated genes and pathways, our study uncovered several novel genes, pathways, and lung cell types influenced by *Adm* in this model.

Conducting RNA-seq studies at various stages of a disease provides insights into the disease’s molecular mechanisms, progression, recovery, and repair. It can also provide insights into potential therapeutic targets and responses to therapies. Since our prior study [48] demonstrated that *Adm* deficiency potentiated LPS-induced lung injury in neonatal mice, the current study aimed to identify potential molecular mechanisms through which *Adm* deficiency causes neonatal lung injury. Therefore, the RNA-seq studies were performed at an early point. Early-stage studies also have the potential to identify biomarkers for early diagnosis of the disease. Future studies will focus on performing high-throughput studies at multiple time points to determine the dynamic processes involved in disease progression and identify stage-specific biological processes and prognostic biomarkers in BPD.

LPS exposure affected the expression of several genes in the experimental BPD model. *Slit2* and *Mmp9* were among the top down-regulated and up-regulated genes, respectively, upon LPS exposure in both *Adm*^+/*−*^ and WT mice. *Slit2*, a member of the Slit-secreted glycoprotein family, acts via its receptor roundabout (Robo)1 to govern cell morphology and movement. In addition to its role in guiding axonal projection and neuronal migration [71,72], *Slit2* is also shown to regulate immune responses, as evident by its prevention of inflammatory cell chemotaxis in the brain [73] and prevention of inflammatory cell recruitment and injury in kidneys [74]. Since inflammatory lung injury is a hallmark of BPD, it is possible that *Slit2* deficiency can lead to BPD phenotype. However, this gene’s pathogenic and biomarker roles in preclinical and clinical BPD are unexplored, highlighting one of the new information provided by our study. In contrast, the finding of increased *Mmp9* expression in our BPD model is supported by a similar finding in other neonatal lung injury models [75,76,77,78] and human BPD [79,80,81], validating our findings’ relevance in BPD.

Notably, the bulk of differentially expressed genes were observed in LPS-exposed *Adm*^+/*−*^ neonatal murine lungs, highlighting the pivotal role of *Adm* signaling in regulating LPS-induced neonatal lung injury. For example, Fibroblast growth factor 10 (*Fgf10*) showed preferential down-regulation upon LPS exposure in *Adm*^+/−^ mice compared to WT mice. *Fgf10* is crucial for lung development, influencing the branching of the embryonic lung [82] and targeting alveolar epithelial progenitor cells and resident mesenchymal cells to control their proliferation and differentiation [83]. It is involved in lung disease and regeneration [84], with protective and therapeutic effects demonstrated in mouse models of lung fibrosis [85] and bronchial regeneration after injury. *Fgf10* expression decreases in the lungs of patients with BPD [86], suggesting its role in the disease’s etiology, and exogenous application of recombinant Ffg10 is being investigated as a potential therapy.

Furthermore, KEGG pathway analysis of genes up-regulated in *Adm*^+/−^ mice without significant change in WT mice revealed significant enrichment of NF-κB and STAT pathways, among others, and these are known to play crucial roles in inflammatory responses. Specifically, the NF-κB signaling pathway is known to regulate the expression of various inflammatory cytokines, chemokines, and adhesion molecules, which are crucial in mediating the inflammatory response in lung tissue [87]. The STAT pathway is integral in transmitting signals for various cytokines and growth factors, contributing to the regulation of immune responses and inflammation [88]. Taken together, these findings suggest that both the NF-κB and STAT pathways are actively involved in the lung inflammatory phenotype observed in our study, potentially explaining the underlying molecular mechanisms.

Notably, Methionine adenosyltransferase (*Mat1a*) was among the top differentially up-regulated genes upon LPS exposure in *Adm*-haplodeficient (*Adm*^+/*−*^) mice. *Mat1a* is an enzyme that catalyzes the formation of S-adenosylmethionine, the primary supplier of methyl groups for most biological methylation reactions [89]. *Mat1a* deficiency causes hypermethioninemia [90]. The enzyme is mainly expressed in the liver, and the liver of the *Mat1a* knockout mice displays markers of increased inflammation, injury, and cellular proliferation [91]. Despite displaying an increased expression of cellular proliferation markers, *Mat1a* knockout mice exhibit attenuated liver regeneration following partial hepatectomy [92]. These findings emphasize the necessity of *Mat1a* in maintaining liver homeostasis. Since lung inflammation and regeneration play important roles in BPD initiation, potentiation, and resolution, *Mat1a* can be one of the major mediators of BPD. Further, increased total and oxidized methionine levels are observed in BPD infants [93,94]. However, whether *Adm* regulates neonatal lung injury via *Mat1a* expression is unexplored. The pathogenic and biomarker roles of *Mat1a* in preclinical and clinical BPD also remain unexplored, highlighting our study’s additional new information. Analysis of major deregulated genes and pathways in LPS-exposed *Adm*-haplodeficient (*Adm*^+/−^) mice indicates the involvement of *Adm* in regulating lung inflammation. Genes regulated by the immune homeostasis modulating transcription factors, Stat1 and Stat3, were significantly up-regulated in the lungs of LPS-exposed *Adm*-haplodeficient (*Adm*^+/−^) mice. These findings hold significant implications for managing BPD since it is a lung disorder of preterm infants characterized by heightened inflammation, a prime driver of disrupted alveolar development.

Several single-cell atlases, specifically of mouse and human, have emerged over the past years as invaluable resources for studying the intricate cellular landscape of individual organs [95]. Pioneering efforts such as the Mouse Cell Atlas [96] and Tabula Muris [56] provide comprehensive insights into the composition and heterogeneity of molecular cell types, thereby providing a tool to help identify key differences in health, infection, and diseases. Similarly, recent endeavors such as the single-cell atlas of the whole human lung have successfully mapped the cellular diversity of human lungs, shedding light on not only lung physiology but also cell type-specific gene expression profiles and regulatory networks key for lung development and homeostasis [97]. These atlas studies leverage cutting-edge technologies to uncover cellular heterogeneity at single-cell resolution, identify rare cell populations, and elucidate gene expression dynamics at an extremely high level of detail, thus laying the groundwork for developing novel therapeutic strategies and personalized medicine approaches in respiratory diseases [98]. Here, we combine findings between bulk RNA sequencing studies and mouse lung atlases [56,57] to determine the role of the *Adm* gene in sepsis-induced neonatal lung injury. We identify key cell types and their function roles in chronic injury and subsequently validate in silico using comparable tracheal aspirate transcriptomic data of extreme premature birth with BPD [63].

The comprehensive integrated bulk-RNA Seq and sc-RNA Seq analyses, incorporating data from our current and published studies, reveal that LPS-exposed *Adm*-haplodeficient (*Adm*^+/−^) murine lungs have increased NK cells and decreased endothelial cells and type II pneumocytes. These results validate the findings of our [44,45,46,48] and other [47] earlier studies, highlighting the protective role of *Adm* in neonatal lung injury since NK cells mitigate inflammation, while endothelial cells and type II pneumocytes promote pulmonary vascularization and alveolarization.

NK cells are innate lymphocytes, constituting up to 15% of resident lung lymphocytes [99]. They regulate innate immunity and potentiate acute injury of the lung and kidney in animal models [100,101,102,103]. Hence, it is conceivable that they can potentiate inflammation and chronic inflammatory injury in neonatal lungs. However, their role in experimental and clinical BPD is understudied. In a two-hit model of experimental BPD incorporating antenatal LPS and postnatal hyperoxia exposure, Zhu et al. [104] observed increased lung NK cells in the BPD group. Contrarily, we did not observe enrichment of NK cell markers in genes that were up-regulated upon LPS exposure in WT mice only. The contrasting results may be due to differences in the timing and number of insults and the severity of lung injury between the two studies. However, LPS-exposed *Adm*-haplodeficient (*Adm*^+/−^) murine lungs in our study demonstrated increased NK cells.

Reconciling our results with those of Zhu et al. [104] once again validates our prior conclusions that LPS-exposed *Adm*-haplodeficient (*Adm*^+/−^) mice have severe neonatal lung injury compared with WT mice [48] since two-hit insults increase lung injury severity compared to single-hit insults [67]. Although we could not find any published evidence of altered NK cell frequency in BPD infants, our in silico analyses using comparable tracheal aspirate transcriptomic data of extremely preterm infants [63] indicate that BPD infants have increased lung NK cells. However, it is unclear if NK cells play a pathogenic role in BPD and if *Adm* regulates neonatal lung injury via NK cells.

Our future studies will address these knowledge gaps. The findings of decreased endothelial cells in the lungs of LPS-exposed *Adm*-haplodeficient (*Adm*^+/−^) mice reconfirm the proangiogenic effects of *Adm* [43,44,45,47,105,106,107,108] and the importance of endothelial cell health in mitigating BPD [12,13,70]. Type II pneumocytes play a vital role in BPD pathogenesis. They are crucial for alveolar development and repair by virtue of their stem cell properties [109,110] and can regulate lung injury severity because of their surfactant-producing properties [111,112,113,114]. Further, they are susceptible to injury from insults such as inflammation and oxidative stress that causes BPD [115,116]. While *Adm* predominantly influences endothelial cell health, it can also affect lung injury by regulating lung epithelial cell proliferation [117] and apoptosis [118]. Therefore, *Adm* can influence neonatal lung injury via its independent and/or interactive effects on NK cells, endothelial cells, and Type II pneumocytes.

Future studies are needed to elucidate the lung cell-specific effects of *Adm* in BPD. Not surprisingly, our in silico analyses using comparable tracheal aspirate transcriptomic data of extremely preterm infants [63] did not show alterations in endothelial and Type II pneumocytes between BPD and non-BPD infants because the tracheal aspirates are primarily composed of secretions from the respiratory tract, including mucus, inflammatory cells, and sometimes bacterial or viral pathogens.

While our study provides significant insights into the affected cell types and molecular mechanisms underlying the observed phenotype, it is important to acknowledge the limitations associated with our reliance on pure in silico analysis. Experimental validation of the key findings, such as functional assays and in vivo and clinical studies, is necessary to confirm the biological relevance and mechanistic roles of the identified genes and pathways. Therefore, future work should focus on integrating our bioinformatic predictions with experimental data to strengthen and validate the conclusions drawn from this study.

## 5. Conclusions

In conclusion, this unbiased, high-throughput study characterizes the gene expression profile of *Adm*-haplodeficient (*Adm*^+/*−*^) mice in an LPS-induced model of experimental BPD and provides molecular and cellular insights through which *Adm* may regulate neonatal lung injury. The study also highlights novel genes and cell types regulated by *Adm* and LPS. Furthermore, our in silico analyses discover that BPD infants have elevated lung NK cells, mirroring the finding in LPS-exposed *Adm*-haplodeficient (*Adm*^+/*−*^) mice. Finally, our findings further validate the protective role of *Adm* in experimental BPD, emphasizing that it is a potential therapeutic target for BPD infants with a predominant inflammatory phenotype.

## Data Availability

Publicly available datasets were analyzed in this study. The data generated for this manuscript can be found here: https://www.ncbi.nlm.nih.gov/geo/query/acc.cgi?acc=GSE156028, accessed on 13 June 2023.

## Supplementary Materials

The following supporting information can be downloaded at: https://www.mdpi.com/article/10.3390/genes15060806/s1, Figure S1: KEGG gene set enrichment analysis; Table S1: Differential expression analysis in WT LPS vs. PBS; Table S2: Differential expression analysis in *Adm* KO LPS vs. PBS; Table S3: Differential expression analysis BPD vs. Control; Table S4: BPD patient characteristics; Table S5: KEGG terms all categories.
