# Supplementary material for: Leveraging Integrated RNA Sequencing to Decipher Adrenomedullin’s Protective Mechanisms in Experimental Bronchopulmonary Dysplasia
Source: Genes (Basel). 2024 Jun 19;15(6):806. doi: 10.3390/genes15060806 (PMC11202456; doi:10.3390/genes15060806)
Supplement: Supplementary file 1 [file genes-15-00806-s001.zip › Figure S1.pdf]

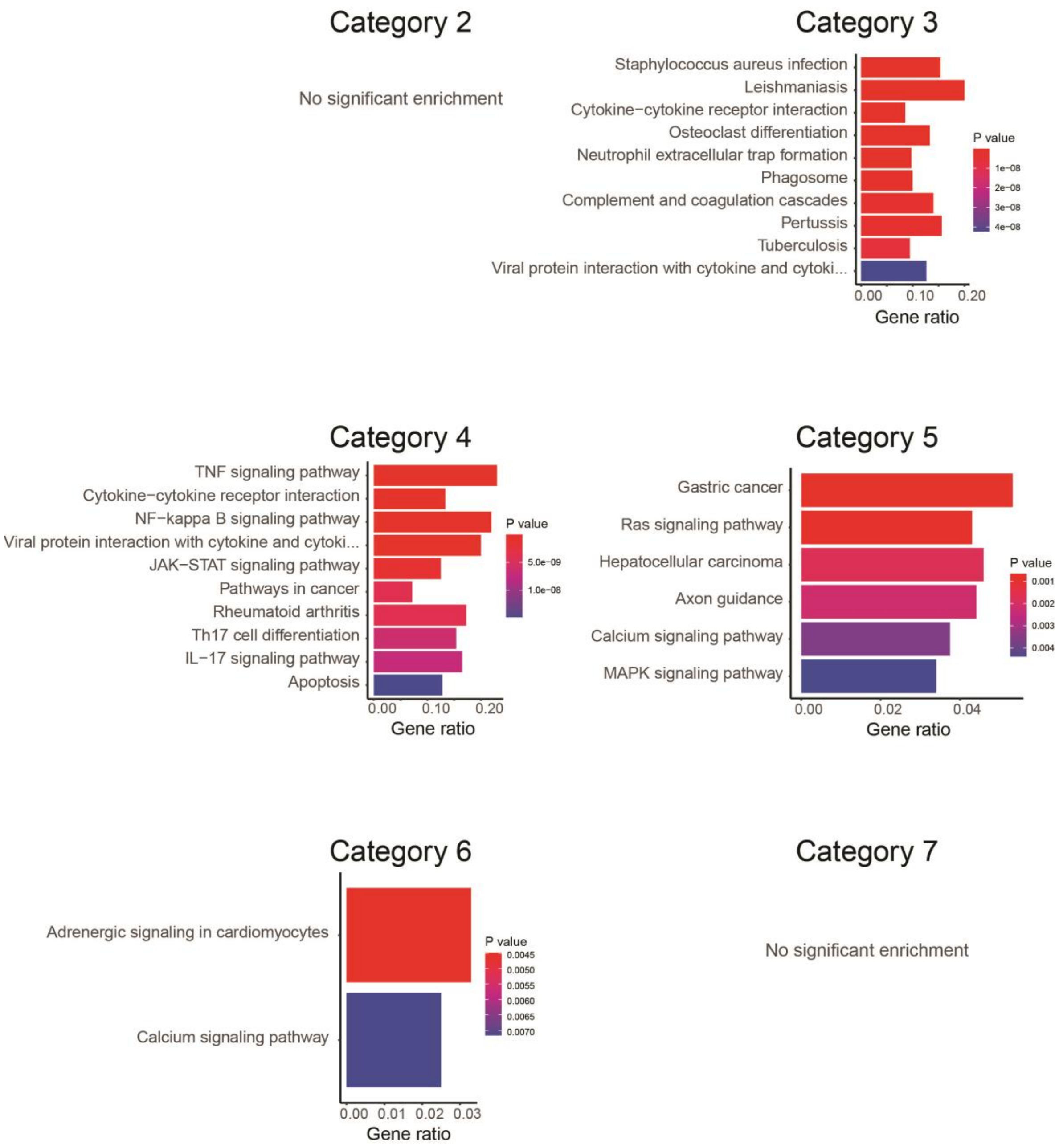

Figure S1. KEGG gene set enrichment analysis. Barplots visualize gene ratio (x-axis) derived from enrichment tests for significant KEGG pathways (y-axis). KEGG pathways were defined as significant if adjusted p-value was less than 0.25 and the gene set contained at least 5 genes. Colors represent statistical significance.
